# Influences of Nursing Work Environment and Patient Safety Environment on Nurse Outcomes

**Published:** 2018-10

**Authors:** Sujin PARK, Youngji KIM

**Affiliations:** 1. Dept. of Nursing, Daegu Health College, Daegu, Republic of Korea; 2. College of Nursing, Gachon University, Incheon, Republic of Korea

## Dear Editor-in-Chief

Nursing has developed remarkably as a professional field. The number of people working in the nursing field has risen as the number of students applying for nursing programs has gradually increased; however, the number of experienced nurses remains insufficient. The nursing work environment should be understood by each country because it varies according to context and culture, but until now, it has been identified by focusing on Western countries (1). The nursing work environment in Korea has not been sufficiently identified. It is believed to influence nurses, but the evidence is insufficient (2).

Patient safety is one of the highest priority goals of nursing care and the importance of the patient safety environment is increasing in the nursing delivery system. Therefore, nurse managers should make patient safety the priority in their hospitals. Current research has directed limited resources toward identifying the patient safety environment, and as such, the impact of the patient safety environment on nurses is not well explicated (3).

This cross-sectional survey was conducted to identify how nurses’ awareness of the nursing work environment and patient safety environment may influence nurses, and the quality of the nursing work environment and patient safety environment are important to nursing retention.

One hundred-thirty respondents (44.5%) perceived their nursing work environment as positive, and 246 (84.2%) perceived their patient safety environment as positive. Nurses’ perceptions of the nursing work environment of respondents were below the cutoff (2.44±0.34), nursing foundations for quality of care were the highest mean score (2.74±0.44), while perceptions of staffing and resource adequacy (2.03±0.53) were the lowest. Nurses’ perceptions of patient safety environment (3.19±0.22) was positive, but their perceptions of their organizational culture attained the lowest mean score (below cutoff). Nurses with unfavorable perceptions of the nursing working environment had a greater likelihood of having higher turnover intention (OR=2.98, CI 1.55–5.72, *P*<.01), lower job satisfaction (OR=0.36, CI 0.14–0.48, *P*<.001) and higher burnout (OR=2.97, CI 1.61–5.46, *P*<.001) compared to those who had favorable perceptions. Nurses with unfavorable perceptions of the patient safety environment had a greater likelihood of having higher turnover intention (OR=3.06, CI 1.16–8.06, *P*<.05), lower job satisfaction (OR=0.37, CI 0.21–0.66, *P*<.01), and higher burnout (OR=2.68, CI 1.48–4.86, *P*<.01) compared to those with favorable perceptions (Table 1). This study found that the nursing work environment was the most significant predictor of the job satisfaction, turnover intention, and burn-out of hospital nurses. There has been no increase in the number of nurses despite the increasing number of nursing students or substitutes because people have not taken full account of the importance of the nursing work environment. Thus, developing strategies through improving the nursing work environment is expected to increase nurse retention. Our findings show that nurses thought they were not able to fully participate in hospital affairs and did not have enough resources and staff. A working environment with a perceived lack of participation in hospital policy and a lack of resources was found to be the main issue in our nursing context (4).

**Table 1:** Logistic regression on nursing work environment, patient safety environment and nurse outcomes

***Predictor***		***Burnout***			***Turnover Intention***			***Job Satisfaction***		
		***Odd Ratio***	***95% CI***	***P***	***Odd Ratio***	***95% CI***	***P***	***Odd Ratio***	***95% CI***	***P***
Nursing work environment	Good	1			1			1		
	Bad	2.98	1.55–5.72	.001	2.97	1.61–5.46	.000	.263	.144–.479	.000
Patient safety environment(Organizational culture)	Good	1			1					
	Bad	3.06	1.16–8.06	.024	2.68	1.48–4.86	.001	.368	.207–.655	.001

* Logistic Regression *P*<.05

Nurses had low satisfaction with their participation in hospital policymaking, and the motivation of nurses to policy making should be encouraged (5). Hospital organizations should make efforts to encourage staff nurses’ participation in the decision-making processes of hospitals and nursing departments. In addition, this study found that patient safety environment was an important predictor for variables related to nursing outcomes such as turnover intention, job satisfaction, and burnout.

Nurses’ perception of safety climates can influence safety performance through their effects on knowledge and motivation (6). Therefore, education for increasing the nurses’ perception on patient safety should be provided to staff nurses in the hospital. Increasing their confidence in patient safety will empower nurses to stay in their current position and increase their satisfaction with their current job. This study highlighted that the organizational culture in the patient safety environment has been identified as having a significant impact on job satisfaction and turnover intention. Traditionally, Korean organization culture was determined as rank oriented and was focused on regulation, rule, and hierarchy (7).
